# *Mycobacterium leprae*-Specific Antibodies in Multibacillary Leprosy Patients Decrease During and After Treatment With Either the Regular 12 Doses Multidrug Therapy (MDT) or the Uniform 6 Doses MDT

**DOI:** 10.3389/fimmu.2018.00915

**Published:** 2018-05-14

**Authors:** Emerith M. Hungria, Samira Bührer-Sékula, Regiane M. Oliveira, Lúcio C. Aderaldo, Maria Araci A. Pontes, Rossilene Cruz, Heitor S. de Gonçalves, Maria L. F. Penna, Gerson O. Penna, Mariane M. A. Stefani

**Affiliations:** ^1^Instituto de Patologia Tropical e Saúde Pública, Universidade Federal de Goiás, Goiânia, Brazil; ^2^Centro de Dermatologia Dona Libânia, Fortaleza, Brazil; ^3^Fundação de Dermatologia Tropical e Venereologia Alfredo da Matta, Manaus, Brazil; ^4^Departamento de Epidemiologia e Bioestatística, Universidade Federal Fluminense, Rio de Janeiro, Brazil; ^5^Núcleo de Medicina Tropical, Universidade de Brasília, e FIOCRUZ Brasília, Brasília, Brazil

**Keywords:** leprosy, serology, phenollic glycolipid-I antigen, LID-1, ND-O-LID, multidrug therapy

## Abstract

Leprosy serology reflects the bacillary load of patients and multidrug therapy (MDT) reduces *Mycobacterium leprae*-specific antibody titers of multibacillary (MB) patients. The *Clinical Trial for Uniform Multidrug Therapy Regimen for Leprosy Patients in Brazil* (*U-MDT/CT-BR*) compared outcomes of regular 12 doses MDT/R-MDT and the uniform 6 doses MDT/U-MDT for MB leprosy, both of regimens including rifampicin, clofazimine, and dapsone. This study investigated the impact of R-MDT and U-MDT and the kinetic of antibody responses to *M. leprae-*specific antigens in MB patients from the *U-MDT/CT-BR*. We tested 3,400 serum samples from 263 MB patients *(*R-MDT:121; U-MDT:142) recruited at two Brazilian reference centers (Dona Libânia, Fortaleza, Ceará; Alfredo da Matta Foundation, Manaus, Amazonas). Enzyme-linked immunosorbent assays with three *M. leprae* antigens [NT-P-BSA: trisaccharide-phenyl of phenollic glycolipid-I antigen (PGL-I); LID-1: Leprosy Infectious Disease Research Institute Diagnostic 1 di-fusion recombinant protein; and ND-O-LID: fusion complex of disaccharide-octyl of PGL-I and LID-1] were performed using around 13 samples per patient. Samples were collected at baseline/M0, during MDT (R-MDT:M1–M12 months, U-MDT:M1–M6 months) and after MDT discontinuation (first, second year). Statistical significance was assessed by the Mann–Whitney *U* test for comparison between groups (*p* values < 0.05). Mixed effect multilevel regression analyses were used to investigate intraindividual serological changes overtime. In R-MDT and U-MDT groups, males predominated, median age was 41 and 40.5 years, most patients were borderline lepromatous and lepromatous leprosy (R-MDT:88%, U-MDT: 90%). The bacilloscopic index at diagnosis was similar (medians: 3.6 in the R-MDT and 3.8 in the U-MDT group). In R-MDT and U-MDT groups, a significant decline in anti-PGL-I positivity was observed from M0 to M5 (*p* = 0.035, *p* = 0.04, respectively), from M6 to M12 and at the first and second year posttreatment (*p* < 0.05). Anti-LID-1 antibodies declined from M0 to M6 (*p* = 0.024), M7 to M12 in the R-MDT; from M0 to M4 (*p* = 0.003), M5 to M12 in the U-MDT and posttreatment in both groups (*p* > 0.0001). Anti-ND-O-LID antibodies decreased during and after treatment in both groups, similarly to anti-PGL-I antibodies. Intraindividual serology results in R-MDT and U-MDT patients showed that the difference in serology decay to all three antigens was dependent upon time only. Our serology findings in MB leprosy show that regardless of the duration of the U-MDT and R-MDT, both of them reduce *M. leprae*-specific antibodies during and after treatment. In leprosy, antibody levels are considered a surrogate marker of the bacillary load; therefore, our serological results suggest that shorter U-MDT is also effective in reducing the patients’ bacillary burden similarly to R-MDT.

**Clinical Trial Registration:**

ClinicalTrials.gov, NCT00669643.

## Introduction

The infection by *Mycobacterium leprae* in humans is characterized by a wide spectrum of clinico-pathological manifestations associated with distinct bacteriologic, immunologic, and histopathologic features categorized as tuberculoid (TT), borderline tuberculoid (BT), borderline borderline (BB), borderline lepromatous (BL), and lepromatous leprosy (LL) (1). In leprosy patients, the specific antibody responses depend on the bacillary load. Vigorous antibody production with low or absent *M. leprae*-specific cell-mediated immunity (CMI) are seen in multibacillary (MB) patients while paucibacillary (PB) leprosy has strong *M. leprae*-specific CMI and low or undetectable antibody levels (2). Over the years, several leprosy serologic tests using different methodologies and antigens have been reported. Lateral flow, dipstick, particle agglutination, and enzyme-linked immunosorbent assays (ELISAs) mostly employing the *M. leprae*–specific native or synthetic di- or trisaccharide epitope of the phenollic glycolipid-I antigen (PGL-I) chemically linked to bovine or human serum albumin *via* octyl or phenyl group (ND-O or NT-P) have been tested in field-based studies (3–9). These studies have shown high IgM positivity in MB patients and low positivity in PB patients (5, 10, 11). After the completion of *Mycobacterium tuberculosis* and *M. leprae* genomes (12, 13), new *M. leprae*-specific proteins have been screened for serology and CMI-tests. The ML0405 and the ML2331 proteins were shown to be highly recognized by MB patients and were later engineered as the di-fusion LID-1 antigen (Leprosy Infectious Disease Research Institute/IDRI Diagnostic-1) (14–16). Positivity to IgG ELISAs to LID-1 is also proportional to the patient’s bacillary load (14, 15, 17, 18). More recently, ND-O-LID antigen, a single fusion complex of natural disaccharide-octyl epitope (ND-O) of PGL-I and LID-1 has been used for the simultaneous, detection of IgM and IgG antibodies in lateral flow test and ELISA (19–23).

Leprosy is a treatable and curable disease and for multidrug therapy (MDT) implementation, patients are classified either as MB (six or more skin lesions, LL, BL, BB forms) or PB (up to five skin lesions, TT and BT forms) (24), The standard leprosy MDT treatment comprises two different regimens: 12 months with rifampicin, dapsone, and clofazimine for MB patients and 6 months with rifampicin and dapsone for PB patients (24). In MB patients, MDT reduces *M. leprae*-specific antibody titers suggesting the application of serology to monitor treatment efficacy (25–34). In 2007, an open-label, randomized clinical trial was conducted to compare the main outcomes [relapses, leprosy reactions, bacilloscopic index (BI) decline] of patients treated with the regular WHO MDT (R-MDT) and a 6-month uniform MDT regimen (U-MDT, rifampicin, dapsone, and clofazimine) for both PB and MB leprosy, regardless of any classification [Clinical Trial for Uniform Multidrug Therapy Regimen for Leprosy Patients in Brazil (U-MDT/CT-BR)] (35–42). As part of the U-MDT/CT-BR, a bank comprising sequential serum samples collected before, during and after R-MDT and U-MDT was assembled. This study, reports the impact of the U-MDT and the R-MDT on leprosy serology to PGL-I, LID-1, and ND-O-LID antigens and the kinetics of antibody responses at different time points in both treatment groups.

## Materials and Methods

### Study Population

Our study group comprises only MB patients from *U-MDT/CT-BR* (Dona Libânia, Fortaleza, Ceará state and Alfredo da Matta, Manaus, Amazonas state), recruited from 2007 to 2015 that had positive bacilloscopy and at least three serum samples collected during monitoring (36). Serum samples tested were collected before MDT (M0/month zero), from 1 to 12 months after the start of MDT (M1–M12) and at the first and second year after the conclusion of treatment (R-MDT first and second year: 24 and 36 months after treatment conclusion, respectively and U-MDT first and second year: 18 and 30 months after treatment conclusion, respectively). Details of patients’ recruitment, diagnosis, and main follow-up outcomes have been previously described (35–43).

### Leprosy Serology

Serologic reactivity to *M. leprae* antigens was assessed by ELISA using the following antigens: natural trisaccharide-phenyl-BSA (NT-P-BSA) a semi-synthetic analog of PGL-I (batch: Nara XVI-61; Dr. Fujiwara, Japan), Leprosy Infectious Disease Research Institute Diagnostic-1 (LID-1) (batch: ago 2012, IDRI, USA) and the single fusion complex (ND-O-LID-batch: 17 August 2012, IDRI, USA).

### Detection of IgM Antibodies to PGL-I

Serum IgM antibodies to PGL-I were detected by ELISA as previously described (3). PolySorp 96-well plates (Nunc, Roskilde, Denmark) were coated with 50 μl/well of 0.01 mg/mL of the sugar component of NT-P-BSA or BSA and blocked with 1% BSA/PBS. Serum samples diluted 1/200 in PBS-Tween containing 10% normal goat serum/NGS (Sigma-Aldrich, St. Louis, MO, USA) were tested in NT-P-BSA and in BSA coated wells. After incubation and washings, horseradish peroxidase/HRP-conjugated anti-human IgM (Immuno Chemicals, St. Louis, MO, USA) was added. In order to control plate-to-plate and day-to-day variation, a positive reference serum was added in duplicate on each plate. After incubation and washings, peroxidase color substrate (TMB, Sigma-Aldrich, St. Louis, MO, USA, Homemade) was added and the reaction was quenched by the addition of 2.5 N H_2_SO_4_, when the OD at 450 nm from reference serum reached an OD value of 0.6. The OD was measured at 450 nm using a Bio-Rad micro plate reader (Life Science, Hercules, CA, USA). The final OD was calculated by subtracting the OD of BSA coated wells from OD values of NT-P-BSA coated wells. The cutoff was defined as OD > 0.25 as previously described (5).

### Detection of IgG Antibodies to LID-1 and Detection of IgM and IgG Antibodies to ND-O-LID

Serum IgG antibodies to LID-1 were detected by ELISA. Polysorp 96-well plates (Corning Costar, NY, USA) were coated with 100 μl/well of 1 µg/mL LID-1 or with 100 μl/well of 0.25 µg/mL ND-O-LID. Blocking was performed with PBS-T 1% BSA. Serum samples diluted 1/200 in PBS-T-10% NGS were added in duplicate and incubated for 2 h at room temperature. Plates were washed and incubated for 1 h with HRP-conjugated anti-human IgG (Southern Biotech, Birmingham, AL, USA) for anti-LID-1 serology or for anti-ND-O-LID serology plates were incubated with anti-human IgG (Southerm Biotech, Birmingham, AL, USA) plus anti-human IgM (Immuno Chemicals, St. Louis, MO, USA). After washings, reactions were developed with peroxidase color substrate (KPL, Gaithersburg, MD, USA) and quenched by the addition of 1 N H_2_SO_4_. The optical density (OD) was determined (Bio-Rad microplate reader, Life Science, Hercules, CA, USA) at 450 nm. For anti-LID-1 serology, the cutoff was calculated as two times the SD of the OD of sera from healthy endemic controls, such that samples with OD > 0.3 were considered positive (15). As previously described, the anti-ND-O–LID serology threshold for positive responses was considered OD >0.923 (20). The results of serologic tests were expressed as the mean OD of duplicates.

### Statistical Analyses

Antibody levels were measured taking into account the medians of the OD at different time points in each treatment group. The percentage of positive samples was calculated based on the number of samples with OD above the cutoff established for each test at each time point. The statistical analyses performed in this study aimed mainly to answer if the data supported the hypothesis that the serological results have a time trend after the beginning of treatment, reflecting the reduction in bacillary load, and if this trend differed between the two treatment groups. The first statistical analysis employed Kruskal–Wallis test one-way analysis of variance for comparison of multiple groups and the Mann–Whitney *U*-test for comparison between two groups comparing data of all patients at different time points. Results were considered statistically significant when *p* values <0.05 were obtained. The intraindividual decay of serology among patients from R-MDT and U-MDT was evaluated employing mixed effects hierarchical/multilevel regression analyses using STATA software (44, 45). The multilevel analyses considered the individual serological results to each different antigen during different time points of follow-up. For these analyses, the independent variable was the serological result, the dependent variables were time and treatment group, and the group variable was patient ID. These analyses allowed the investigation of the effects that vary by group (each patient) and estimate group level averages in which each patient has his own time trend where one measure is not independent of the previous one.

## Results

### Main Characteristics of Study Population

In this study, we have assessed the serologic reactivity of 3,400 sequential serum samples collected at different time points, from 263 MB leprosy patients, with positive bacilloscopy, enrolled at U-MDT/CT-BR and treated either with the R-MDT or U-MDT regimens. Among 263 MB patients, 56 were from Amazonas State and 207 came from Ceará State. In our study group, 54% (142 out of 263) received U-MDT and 46% (121 out of 263) were treated with R-MDT. For each patient, a median of 13 sequential serum samples (range: 3–21 samples) was collected at different time points: before MDT (M0), during treatment (M1–M6 for U-MDT and M1–M12 for R-MDT) and after treatment conclusion (first and second year). The main clinical and laboratory features of MB leprosy patients included in this serological study were similar (Table 1). The majority of MB leprosy patients was male, and patients from R-MDT and U-MDT groups had similar age (median age: 41 and 40.5 years, respectively). The majority of patients from the R-MDT and U-MDT groups was classified as BL and LL leprosy (R-MDT: 88%, 107 out of 121; U-MDT: 90%, 128 out of 142). The median of the BI in the R-MDT group was 3.6 (0.2–5.75 range) and 3.8 (0.2–6 range) in the U-MDT group. In the R-MDT group, 61% (69 out of 113) developed a reactional episode, 67% had reversal reaction (RR) (46 out of 69) of these, 11% (5 out of 46) at diagnosis and 89% during follow-up (41 out of 46). In the R-MDT, 33% (23 out of 69) had erythema nodosum leprosum/ENL, of these 4% (1 out of 23) at diagnosis and 96% (22 out of 23) during follow-up. In the U-MDT group, 62% was reactional (82 out of 132) of these 72% had RR (59 out of 82) of these, 14% (8 out of 59) at diagnosis and 86% during follow-up (51 out of 59). In the U-MDT, 28% (23 out of 82) had ENL, of these 9% (2 out of 23) at diagnosis and 91% (21 out of 23) during follow-up.

**Table 1:** Main clinical and laboratory characteristics of the 263 MB leprosy patients enrolled at U-MDT/CT-BR stratified according to the treatment group.

	R-MDT (*n* = 121)	U-MDT (*n* = 142)
Gender (male/female)	86/35	105/37
Age (years) median (range)	41 (8–65)	40.5 (7–65)
R&J classification	12 BT, 2 BB, 75 BL, 32 LL	9 BT; 5 BB; 89 BL, 39 LL
BI median (range)	3.6 (0.2–5.75)	3.8 (0.2–6)
Development of reactions	73/121 (60%)	88/142 (62%)
Type and moment of development of reactions	RR: 50/73	RR: 62/88
	At diagnosis: 7/50	At diagnosis: 8/62
	During follow-up: 43/50	During follow-up: 54/62
	ENL: 23/73	ENL: 26/88
	At diagnosis: 1/23	At diagnosis: 2/26
	During follow-up: 22/23	During follow-up: 24/26

*R&J, Ridley and Jopling classification system; BB, borderline borderline; BL, borderline lepromatous; LL, lepromatous leprosy; BI, bacilloscopic index; R-MDT, regular multidrug therapy; U-MDT, uniform multidrug therapy; RR, reversal reaction; ENL, erythema nodosum leprosum; MDT, multidrug therapy; BT, borderline tuberculoid*.

### Decline of Anti-PGL-I, Anti-LID-1, and Anti-ND-O-LID *M. leprae*-Specific Antibody Levels in the U-MDT and R-MDT Groups During Follow-Up

**Figure 1:**
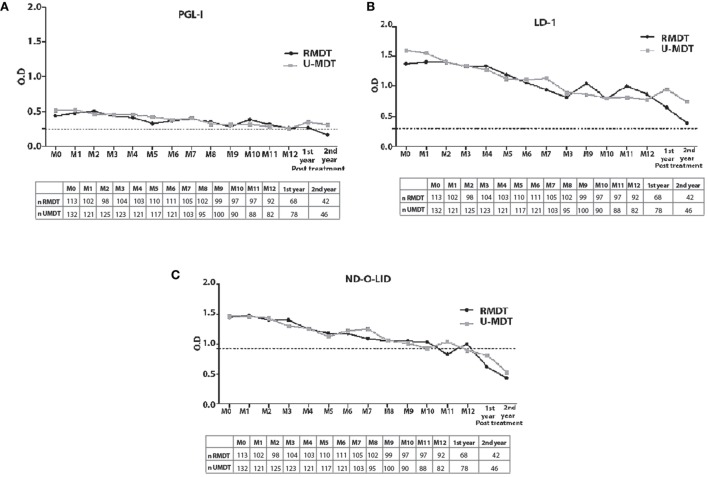
Kinetic of *Mycobacterium leprae*-specific antibody responses in multibacillary treated with regular multidrug therapy (R-MDT) (closed black circles) and uniform multidrug therapy (U-MDT) (gray closed squares) from baseline month zero (M0) to month 12 (M12) after starting treatment, and after the first and second year of treatment conclusion. **(A)** Anti-phenollic glycolipid-I antigen serology; **(B)** anti-LID-1 serology; **(C)** anti-ND-O-LID serology. Each point represents the median optical density (OD) value of each group. The dotted horizontal lines indicate cutoff points of each serological test.

Compared to baseline results, in both R-MDT and U-MDT groups, a significant decline in anti-PGL-I levels was observed upon treatment (Figure 1A). At baseline, the median OD in the R-MDT group was 0.437 and in the U-MDT group, it was 0.516; after 5 months of treatment, the median OD was 0.325 in the R-MDT group and 0.424 in the U-MDT group (*p* = 0.035 and *p* = 0.04, respectively). In the R-MDT group considering baseline (M0) serology, there was a significant decline of the anti-PGL-I levels in the subsequent months (M0 vs M5, *p* = 0.03; M0 vs M7, *p* = 0.09; M0 vs M8, *p* = 0.01; M0 vs M9, *p* = 0.01; M0 vs M10, *p* = 0.02; M0 vs M11, *p* = 0.007; M0 vs M12, *p* = 0.001, M0 vs first year, *p* = 0.02; M0 vs second year, *p* < 0.0001) (Figure 1A). In the U-MDT group, anti-PGL-I levels at diagnosis also reduced during and after treatment (M0 vs M5, *p* = 0.04; M0 vs M6, *p* = 0.02; M0 vs M7, *p* = 0.01; M0 vs M8, *p* = 0.001; M0 vs M9, *p* = 0.003; M0 vs M10, *p* = 0.0007; M0 vs M11, *p* = 0.001; M0 vs M12, *p* < 0.0001, M0 vs first year, *p* = 0.03; M0 vs second year, *p* = 0.0004) (Figure 1A).

Regarding serological results in the R-MDT group, a significant decline in IgG anti-LID-1 antibodies was observed comparing baseline and M6 (median ODs at M0 = 1.386 vs M6 = 1.068; *p* = 0.024) (Figure 1B). In the R-MDT group, anti-LID-1 antibodies continued to decrease during subsequent months (M0 vs M7, *p* = 0.0003; M0 vs M8, *p* < 0.0001; M0 vs M9, *p* = 0.0009; M0 vs M10, *p* < 0.0001; M0 vs M11, *p* = 0.0005; M0 vs M12, *p* < 0.0001), and at the first and second year posttreatment (M0 vs first year, *p* = 0.002; M0 vs second year, *p* < 0.0001) (Figure 1B). In the U-MDT group, anti-LID-1 antibodies declined significantly from baseline to the fourth month of treatment (median ODs at M0 = 1.605, at M4 = 1.279; *p* = *p* = 0.003). Compared to baseline data/M0, anti-LID-1 antibodies decreased after M5 (M0 vs M5, *p* < 0.0001; M0 vs M6, *p* < 0.0001; M0 vs M7, *p* = 0.0003 and M0 vs M8, M0 vs M9, M0 vs M10, M0 vs M11 and M0 vs M12, *p* < 0.0001) and at the first and second year posttreatment (M0 vs first year and M0 vs second year, *p* < 0.0001) (Figure 1B).

In the R-MDT group, serology using ND-O-LID antigen showed a significant decline in antibody levels from baseline (M0) to the seventh month of treatment (M7) (median ODs at M0 = 1.449 vs M7 = 1.092; *p* = 0.005) (Figure 1C). Among patients treated with R-MDT, anti-ND-O-LID antibodies continued to decrease after M8 (M0 vs M8, *p* = 0.003; M0 vs M9, *p* = 0.002; M0 vs M10, *p* = 0.0007; M0 vs M11, *p* = 0.0006; M0 vs M12, *p* < 0.0001, M0 vs first year and M0 vs second year posttreatment, *p* < 0.0001) (Figure 1C). In the U-MDT group, a significant decline in anti-ND-O-LID antibodies was observed from baseline to the fifth month (median ODs M0 = 1.466 vs M5 = 1.126; *p* = 0.006) and antibody levels decreased after M6 (M0 vs M6, *p* = 0.02; M0 vs M7, *p* = 0.01; M0 vs M8, *p* = 0.0006; M0 vs M9, *p* = 0.0003; M0 vs M10, *p* = 0.0001; M0 vs M11, *p* = 0.0006 and M0 vs M12, *p* < 0.0001) and in the first year and second year posttreatment (*p* < 0.0001) (Figure 1C).

### Decline in the Positivity Rates for Anti-PGL-I, LID-1, and ND-O-LID Antibodies Among U-MDT and R-MDT Groups

**Figure 2:**
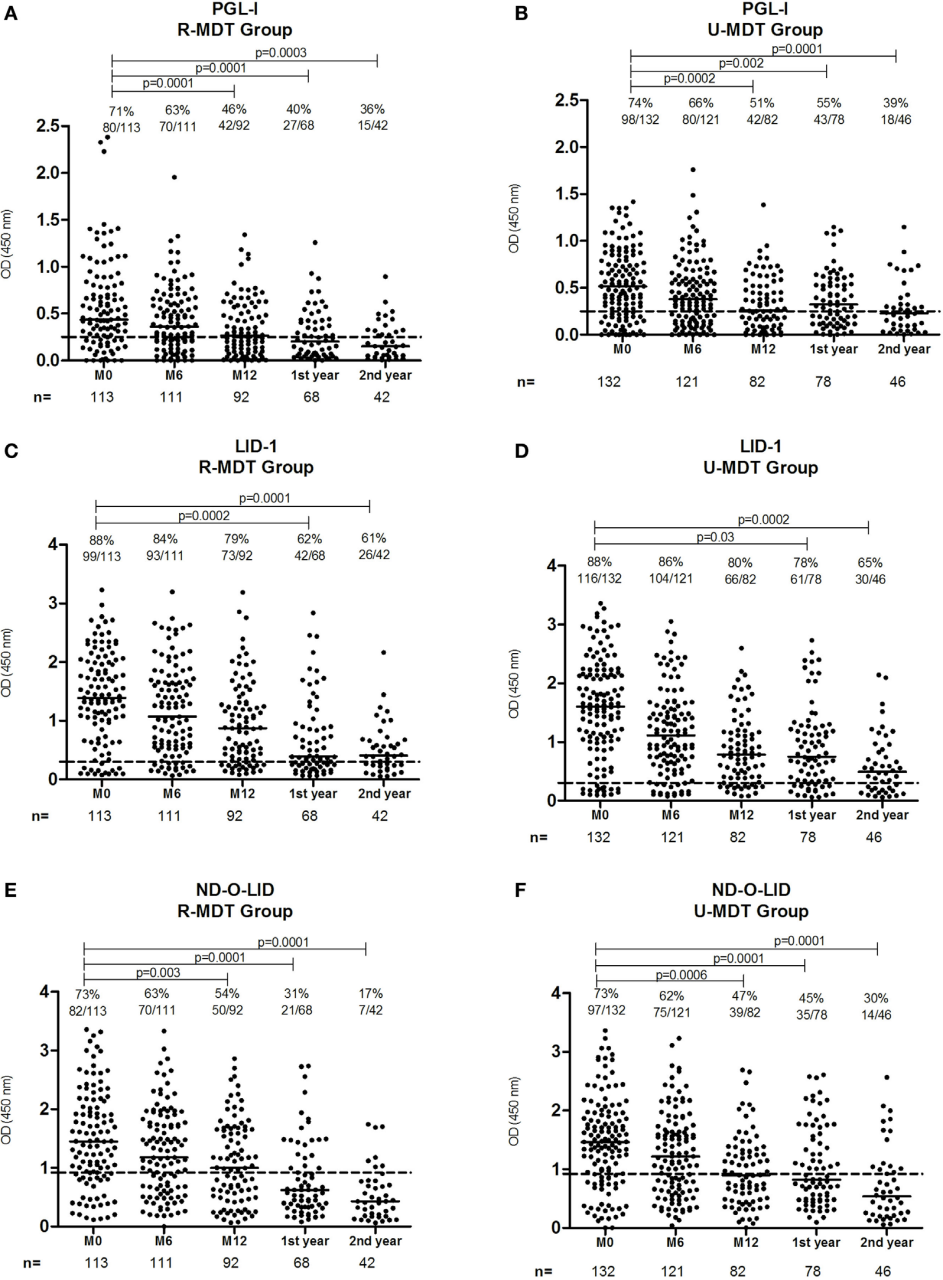
Serologic reactivity to phenollic glycolipid-I antigen (PGL-I), LID-1, and ND-O-LID antigens among multibacillary patients from the regular multidrug therapy (R-MDT) and uniform multidrug therapy (U-MDT) groups at different time points MO, M6, M12, and first and second year posttreatment: anti-PGL-I positivity rates in **(A)** R-MDT group, **(B)** U-MDT group; anti-LID-1 positivity rates in **(C)** R-MDT group, **(D)** U-MDT group; anti-ND-O-LID positivity rates in **(E)** R-MDT group, **(F)** U-MDT group. Each point represents the mean optical density (OD) of duplicates of each individual patient. The median OD value of each group is represented by the horizontal line and the traced line represents the different cutoffs (PGL-I OD > 0.25, LID-1 OD > 0.3, and ND-O-LID OD > 0.923). The number above each dataset is the percent of positive responses. The *p* value refers to differences in positivity rates at different time points.

At baseline, 71% of MB patients who received R-MDT was anti-PGL-I positive, after 6 months MDT (M6) positivity declined to 63% (*p* > 0.05), and at the end of treatment (M12) 46% of patients remained anti-PGL positive (M0 vs M12, *p* = 0.0001) (Figure 2A). In the first-year posttreatment, 43.5% (27 out of 62) was positive and in the second-year posttreatment, 38% (15 out of 39) remained positive. Regarding baseline serology, the decline in anti-PGL-I positivity in the R-MDT group was statistically significant (M0 vs M12, M0 vs first year, M0 vs second year posttreatment, *p* = 0.0001).

In the U-MDT group, 74% was anti-PGL-I positive at baseline, 66% at 6 months (M0 vs M6, *p* > 0.05) and at M12, 51% remained anti-PGL-I positive (M0 vs M12, *p* = 0.0002) (Figure 2B). Positivity rate in the first-year posttreatment was 58% (43 out of 74) and in the second-year posttreatment 44% (18 out of 41) remained positive. Compared to serology at diagnosis, in the U-MDT group, the reduction in anti-PGL-I positivity rate was statistically significant (M0 vs first year, *p* = 0.008; M0 vs second year posttreatment, *p* = 0.0001). Anti-PGL-I positivity rates in patients from the R-MDT and the U-MDT regimens were similar at different time points: M0, M6, M12, and second year posttreatment (*p* > 0.05) (Figures 2A,B). In the first-year posttreatment, anti-PGL-I positivity rate was higher in the U-MDT than in the R-MDT group (*p* = 0.04).

In the R-MDT group, anti-LID-1 positivity rate at baseline was 88%, after 6 months of treatment (M6) 84% remained positive and at the end of MDT (M12), 79% had antibodies above the positivity threshold (Figure 2C). In the first year posttreatment, 62% (42 out of 68) of patients was seropositive and in second year 61% (26 out of 42) remained positive. In the R-MDT group, the percentage of anti-LID-1 positivity was similar at M0, M6, and M12 (*p* > 0.05). However, a statistically significant reduction in positivity rate was seen comparing M0 and first year (*p* = 0.0002) and M0 and second year posttreatment (*p* = 0.0001). In the U-MDT group, before treatment 88% of the patients was anti-LID-1 positive, after 6 months/end of treatment 86% was positive and at M12 80% remained positive (Figure 2D). In first year posttreatment, 78% (61 out of 78) was anti-LID-1 positive and in second year positivity was 65% (30 out of 46). The positivity rate to LID-1 antigen was similar at different time points: M0, M6, and M12 (*p* > 0.05). The reduction in the positivity rate to LID-1 serology was significant comparing M0 vs first year and M0 vs second year posttreatment (*p* = 0.03 and 0.0002, respectively). Positivity rates to LID-1 serology between U-MDT and R-MDT groups were similar at M0, M6, and M12 (*p* > 0.05) (Figures 2C,D). In the first year posttreatment, a statistically significant difference was observed between U-MDT and R-MDT regimens (*p* = 0.01).

Anti-ND-O-LID positivity in the R-MDT group was 73% at baseline, after 6 months MDT (M6) positivity was 63% (*p* > 0.05) and at the end of treatment (M12), 54% of patients remained positive (M0 vs M12 *p* = 0.003) (Figure 2E). At the first year posttreatment, 31% (21/68) of patients remained positive and in the second year 17% were seropositive (7 out of 42) (M0 vs first year, *p* = 0.0001, M0 vs second year, *p* = 0.0001). In U-MDT group, before treatment, 73% of the patients was anti-ND-O-LID positive, after 6 months/end of treatment, positivity was 62% and at M12, 47% remained positive (Figure 2F). In the U-MDT group, the decrease in anti-ND-O-LID positivity rate during the first 12 months of monitoring was statistically significant (M0 vs M12, *p* = 0.0006). There was a significant decline in the positivity rate to anti-ND-O-LID serology from baseline to the first year posttreatment and from baseline to the second year after treatment conclusion (M0 vs first year, *p* = 0.0001; M0 vs second year, *p* = 0.0001). Anti-ND-O-LID positivity rates between U-MDT and R-MDT were similar at M0, M6, and M12 (*p* > 0.05) (Figures 2E,F). In the first year after treatment conclusion, a statistically significant difference in positivity rate was observed between U-MDT and R-MDT regimens (*p* = 0.03).

Our study group of 263 MB patients included the BT, BB, BL, and LL categories, according to the adapted Ridley and Jopling classification system used. Among 263 MB patients, 28 were either BT (*n* = 14) or BB (*n* = 14), representing 12% of the R-MDT group and 10% of the U-MDT group. BT and BB have lower BI compared to BL and LL categories and as serology reflects the BI of the patient, the impact of BT/BB patients on the serology was analyzed by removing these 28 patients from the groups and by comparisons of results of the groups with and without BT/BB. Our results showed that difference in serological results obtained upon exclusion of BT and BB patients was statistically significant only for LID-1 serology at M6 while for all other antigens and time points there was no statistically significant change in the positivity rate by comparing the whole group of BT, BB, BL, LL, and the group of BL and LL patients (Table S1 in Supplementary Material).

**Figure 3:**
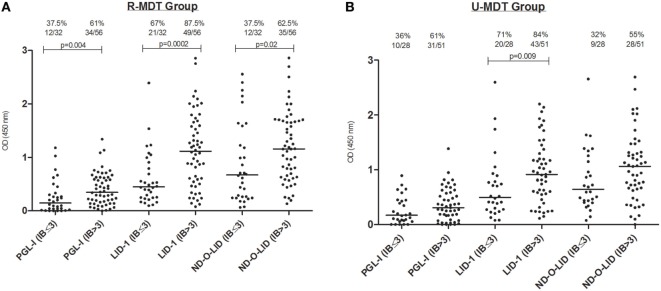
Serologic reactivity at 12 months to phenollic glycolipid-I antigen (PGL-I), LID-1, and ND-O-LID antigens among multibacillary (MB) patients stratified by BI ≤ 3 and BI > 3 from the regular multidrug therapy (R-MDT) **(A)** and uniform multidrug therapy (U-MDT) **(B)** groups. Each point represents the mean OD of duplicates of each individual patient. The median OD value of each group is represented by the horizontal line and the traced line represents the different cutoffs (PGL-I OD > 0.25, LID-1 OD > 0.3, and ND-O-LID OD > 0.923). The number above each dataset is the percent of positive responses. The *p* value refers to differences in medians at different time points. OD, optical density; BI, bacilloscopic index.

The association between the BI at diagnosis and the serologic responses to the three antigens was evaluated 12 months after initiation of both regimens (Figures 3A,B). In the R-MDT group, these analyses included 32 patients with BI ≤ 3 (median = 2, range 0.2–3.0) and 56 patients with BI > 3 (median = 4, range 3.2–5.75). For all antigens tested, the median of OD at M12 was higher among patients with initial BI > 3 compared to patients with BI ≤ 3 (*p* < 0.05) especially for LID-1 serology (*p* = 0.0002) (Figure 3A). In the U-MDT group, 28 patients with BI ≤ 3 (median BI = 1.775, range 0.2–3) and 51 patients with BI > 3 (median BI = 4, range 3.2–6) had their serologic responses compared at M12. In this group, a higher OD value to all three antigens was seen in patients with BI > 3, however, reaching statistical significance only for LID-1 antigen (*p* = 0.009) (Figure 3B).

### Intraindividual Decline of Anti-PGL-I, Anti-LID-1, and Anti-ND-O-LID *M. leprae*-Specific Antibody Levels in the U-MDT and R-MDT Groups

**Figure 4:**
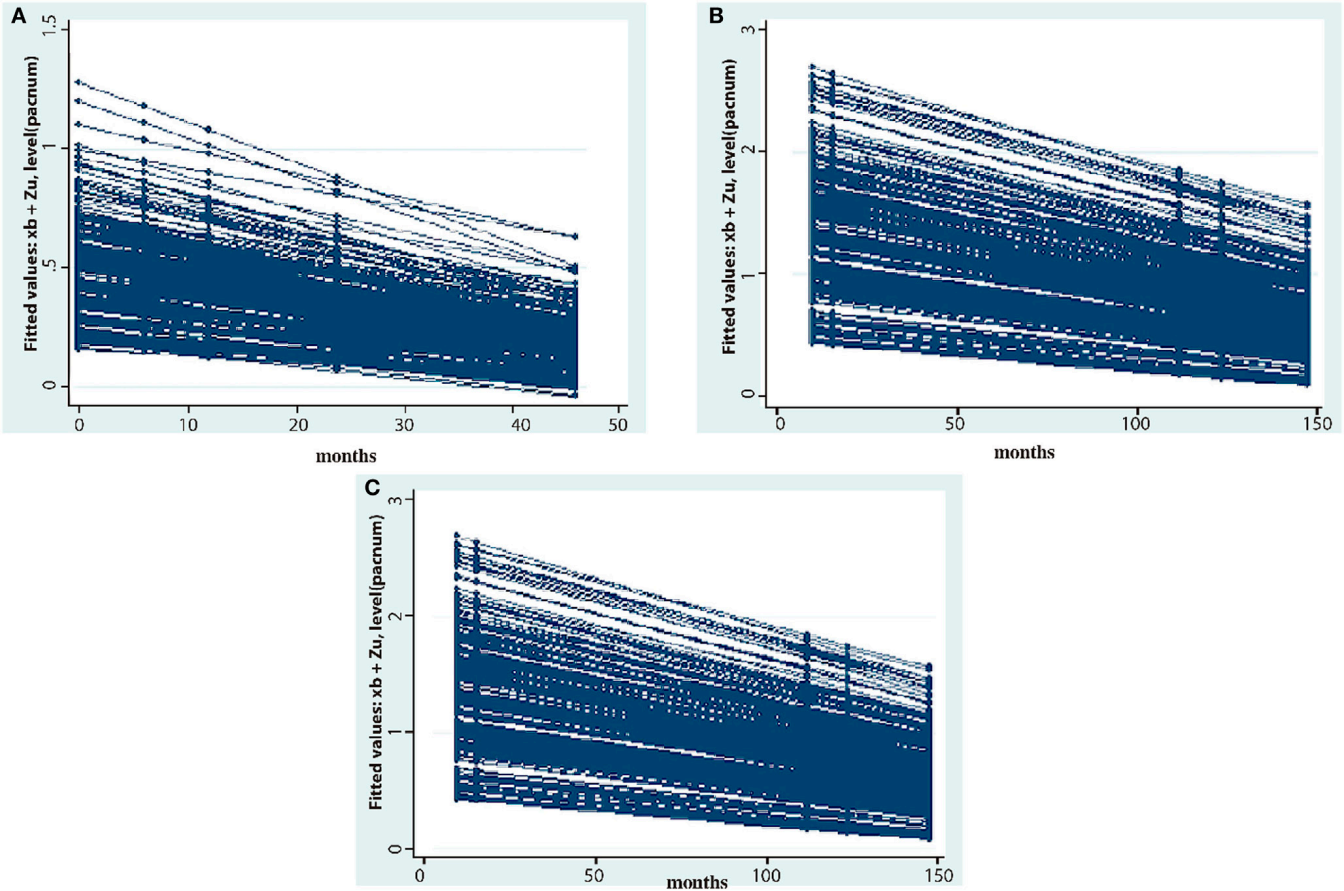
Mixed effect multilevel analyses of 244 patients treated either with regular multidrug therapy (R-MDT) or the uniform multidrug therapy (U-MDT) showing the decay of antibody responses to different *Mycobacterium leprae* antigens (optical density, *y* axis) overtime (months, *x* axis): **(A)** phenollic glycolipid-I antigen, **(B)** LID-1, **(C)** ND-O-LID.

Multilevel regression analyses were performed with serologic results to PGL-I (Figure 4A), LID-1 (Figure 4B), and ND-O-LID antigens (Figure 4C) at different time points of follow-up in 850 samples of 244 patients from both R-MDT and U-MDT groups. These analyses showed that the difference in serologic decay to all three antigens was dependent upon time only. Similar decay of serology to all three antigens was seen among patients from both R-MDT and U-MDT (Figures 4A–C; Table 2).

**Table 2:** Results of adjusted mixed multilevel regression analyses of anti-PGL-I, anti-LID-1, and anti-NDO-LID serology overtime in leprosy patients treated with R-MDT and U-MDT.

	Coefficient	SE	*z*	*p* > *z*	95% Confidence interval	
**Anti-PGL-I**						
Month	−0.006871	0.0007285	−9.43	0	−0.0082988	−0.0054431
Group	−0.0035163	0.0333417	−0.11	0.916	−0.0688648	0.0618322
_Cons	0.5085021	0.1160942	4.38	0	0.2809617	0.7360426
**Anti-LID-1**						
Month	−0.0051023	0.0003361	−15.18	0	−0.005761	−0.0044436
Group	−0.0571696	0.0719866	−0.79	0.427	−0.1982608	0.0839216
_Cons	1.618.408	0.2525812	6.41	0	1.123.358	2.113.458
**Anti-NDO-LID**						
Month	−0.0164264	0.0012887	−12.75	0	−0.0189521	−0.0139006
Group	0.0016501	0.074866	0.02	0.982	−0.1450847	0.1483848
_Cons	1.379.876	0.2617582	5.27	0	0.866839	1.892.912

*The mixed effects multilevel regression analyses evaluated the individual results of anti-PGL-I, anti-LID-1, and anti-ND-O-LID serology during follow-up considering the serological result as the independent variable (constant), the dependent variables were time (month) and treatment group (R-MDT and U-MDT) and the group variable was the patient ID. These analyses showed that the difference in serologic decay to all three antigens was dependent upon time only*.

*PGL-I, phenollic glycolipid-I antigen; R-MDT, regular multidrug therapy; U-MDT, uniform multidrug therapy*.

## Discussion

This study including cross sectional and intraindividual analyses showed that both shorter 6 months U-MDT and standard 12 months R-MDT using rifampicin, dapsone, and clofazimine had a similar effect on leprosy specific serology, reducing the antibodies of MB leprosy patients to three well-characterized *M. leprae* antigens: PGL-I, LID-1, and anti-ND-O-LID. Serologic responses detected at baseline declined during the course of therapy and continued to decline after discontinuation of specific treatment. Multilevel analyses of intraindividual responses showed that for both treatment regimens R-MDT and U-MDT, the decay of serologic reactivity to all three antigens tested was dependent on time only. In leprosy, serology is considered a surrogate marker of the bacillary load and a previous study has shown that MB patients from the R-MDT and the U-MDT groups had similar reduction in the bacillary load (39). In both treatment groups, despite minor oscillations, the pattern of decline in antibody levels was similar for all three *M. leprae* antigens tested. Our results indicate that, regardless of the duration of the treatment regimens for multibacillary patients, antibodies decline overtime during and after treatment interruption. This is the first description of the dynamics of antibody responses to three *M. leprae*-specific antigens among a well-characterized cohort of MB patients, mostly with high bacillary load at diagnosis, who was treated with two different MDT regimens and rigorously monitored during a clinical trial in Brazil. Our study sample contained a robust collection of 3,400 serum samples, including around 13 sequential samples per patient, which were collected since diagnosis over a 3-year period and this serologic study revealed the kinetic of specific antibody responses during this period.

The decrease in antibody levels following MDT, especially to PGL-I, has been well reported in previous studies showing longitudinal data (25–32, 34). In the U-MDT group, antibody titers to all three antigens tested, decreased during the 6-month treatment and despite some variation, anti-PGL-I antibodies continued to decline after treatment discontinuation until the first and second year after MDT conclusion. However, among MB patients most of them with high bacillary load at diagnosis, despite the decline in antibody levels, most patients remained seropositive 2 years after treatment conclusion. The decline in anti-PGL-I positivity from baseline to 2 years after treatment discontinuation was similar in both treatment groups when 38% in the R-MDT and 44% in the U-MDT showed serological responses above the cutoff for positivity. A study among MB Venezuelan patients with high BI showed that the levels of anti-PGL-I antibodies dropped by 57% over 2 years (30). In general, previous studies have shown 50–90% drop in anti-PGL-I levels 2 years after conclusion of treatment (31, 46, 47). Analyses of antibody responses to different antigens in three MB patients showed little, if any, decline in anti-PGL-I serology after MDT (33). Overall, our results show that the gradual drop in antibody levels seen in both U-MDT and R-MDT groups is consistent with a slow reduction in the bacillary load in both treatment groups. In fact, the decline in BI in MB patients is known to occur slowly (0.5–1 log U/year), so that a significant proportion of patients with very high BI at diagnosis, independently of therapy duration, may remain slit skin smear positive for years after treatment (24), therefore stimulating antibody production.

In our study, the percentage of seropositivity varied among tests and also at different time points probably reflecting the sensitivity of the test and the initial bacillary load of the patients. Our data showed that anti-LID-1 serology provided the highest positivity rate at baseline, but as discussed, the decrease in seropositivity was gradual. Among MB patients, the positivity rates remained high 2 years posttreatment, especially to LID-1 antigen. Our results differ from earlier studies that have shown a faster decline in anti-LID-1 titers compared to anti-PGL-I (32, 33). These previous results have suggested that protein antigen was cleared faster than glycolipid/carbohydrate PGL-I antigen. A study showed that a single dose of rifampicin caused a rapid fall in the PGL-I antigen in serum of untreated MB patients (29) and the extent of the reduction of the PGL-I antigen in antibody production is suggested by decreased serology. However, in LL Brazilian patients, the anti-PGL-I antibody levels pre and posttreatment showed a small drop in positivity (from 100 to 90%) and also in BL patients (from 100 to 80%) (32). Results obtained among Venezuelan cured patients who were evaluated 10 years after treatment showed very low anti-LID-1 levels (32). Also, similar to our findings, in MB patients with high bacillary load, the anti-LID-1 positivity rates pre and posttreatment were either unchanged or slightly reduced: from 87 to 91% in LL and from 100 to 79% in BL patients (32). In another study among Brazilian patients, antibody levels to PGL-I and LID-1 dropped after MDT conclusion, however, some patients remained positive around 2 years after MDT (34). In the current study, non-compliance to MDT can be excluded among the causes for persistent antibody production as all patients were fully compliant to R-MDT and U-MDT; however, the high bacillary burden at diagnosis is compatible with a longer time required for the complete clearance of bacillary antigens. In fact, our results showed that despite the consistent reduction in antibody levels to all three antigens investigated, this decrease was gradual over time, so that 2 years after the conclusion of treatment, a significant percentage of patients continued to be seropositive. In conclusion, for MB patients with high BI at diagnosis, 2 years follow-up after treatment conclusion seems a short period for a significant clearance of antigens and also for a significant decrease in seropositivity. Therefore, the applicability of serology to monitor treatment efficacy seems limited for MB patients with high bacillary load at diagnosis evaluated in a short-term follow-up after treatment conclusion, such as 2 years.

The loss to follow-up in the first and second year posttreatment represents a limitation of the current study, however, the consistent and gradual decline in antibody levels seen overtime for all three antigens investigated indicates the validity of our data. Our results showed that the decline patterns seen in anti-PGL-I and anti-ND-O-LID serology were very similar. The simultaneous detection of IgM and IgG to the ND-O-LID conjugate which contains the disaccharide epitope of PGL-I and the diffusion LID-1 protein did not enhance sensitivity. A recent study on leprosy patients showed similar high positivity with these three antigens in BL and LL patients while the proportion of seropositivity to PGL-I and anti-ND-O-LID antigens was similar but lower than anti-LID-1 positivity (48). Another recent study which evaluated the diagnostic potential of two rapid tests using different antigens (PGL-I and ND-O-LID) and technologies (immunochromatographic lateral flow and luminescent-up-converting phosphor UCP-LFA) showed that both tests corresponded to BI but the UCP-LFA showed higher sensitivity (49). The use of ND-O-LID conjugate antigen in leprosy serology, which is expected to enhance sensitivity is recent and results reported so far are not conclusive if detection of IgM and IgG antibodies to the antigens contained in ND-O-LID effectively leads to higher sensitivity than observed using individual antigens and this topic deserves further investigations.

Previous studies have investigated the potential use of leprosy serology as a marker of reactional episodes (50–52). Two previous publications from our group using the U-MDT database and sera bank (40, 41) have described the impact of baseline serology on the development of leprosy reactions. The baseline ML flow test results showed limited sensitivity and specificity as prognostic markers for the development of leprosy reactions during subsequent follow-up (40). Also, the analyses of anti-PGL-I, anti-LID-1, anti-ND-O-LID antibodies at diagnosis showed low sensitivity and specificity for predicting reversal reaction while anti-LID-1 serology at diagnosis showed prognostic value for the development of ENL in BI positive patients (41). We acknowledge the importance of the analysis of the impact of reactional episodes on longitudinal serology data, however, these analyses are out of the scope of the current study which focused on the effect of different treatment regimens on leprosy serology to three antigens. Leprosy serology reflects the bacillary load of the patient and several studies have reported that MB patients are more vulnerable to develop leprosy reactions. In this sense, slit skin smears at diagnosis can indicate patients at higher risk of developing leprosy reactions, but these tests are not part of diagnostic routine and are not used to monitor reactions or relapse. Compared to slit skin smears, serology can be considered a simpler test that could indicate the risk for the development of reactions, especially ENL.

In conclusion, our study on MB leprosy patients, the majority with high bacillary load at diagnosis, indicated a similar decrease in *M. leprae* antibody production to PGL-I, LID-1, and ND-O-LID in patients treated with R-MDT and U-MDT for 12 and 6 months, respectively. The slow reduction in seropositivity rates seen in MB patients treated with both R-MDT and U-MDT is compatible with the slow decline of bacillary load, regardless of the duration of the treatment. This slow reduction indicates that the applicability of serological monitoring to evaluate MDT efficacy or track the effectiveness of MDT is limited at least in short term period of 2 years posttreatment as within this time period, a significant rate of patients remains positive.

## Ethics Statement

This study was carried out in accordance with the recommendations of the Resolution 466/2012 from the National Health Council/Ministry of Health, with written informed consent from all subjects. All subjects gave written informed consent in accordance with the Declaration of Helsinki. The protocol was approved by the National Committee for Ethics in Research (CONEP) (protocol number 001/06). Data confidentiality was strictly guaranteed and all patients were free to leave the study and opt for the R-MDT regimen outside the study (ClinicalTrials.gov identifier: NCT00669643).

## Author Contributions

EH, SB-S, RO, GOP, and MMAS conceived and designed the experiments. EH, SB-S, RO, and MMAS performed the experiments. EH, SB-S, MLFP, GOP, and MMAS analyzed the data. SB-S, LA, MAAP, RC, HG, MLFP, GOP, and MMAS contributed with reagents/materials/analysis tools. EH, SB-S, and MMAS wrote the paper. EH, SB-S, and MLFP were in charge of data bank preparation and quality control of data. EH, SB-S, LA, RO, MAAP, HG, MLFP, GOP, and MMAS participated in the interpretation of data, critical reading and approval of the final manuscript. We thank Carlos Sarina, FIOCRUZ, Brasilia for providing high resolution figures.

## Conflict of Interest Statement

The authors declare that the research was conducted in the absence of any commercial or financial relationships that could be construed as a potential conflict of interest. The reviewer AV declared a shared affiliation with the handling Editor, and the reviewer RP declared a shared affiliation, though no other collaboration, with one of the authors GP.
