# Supplementary material for: Mycobacterium leprae-Specific Antibodies in Multibacillary Leprosy Patients Decrease During and After Treatment With Either the Regular 12 Doses Multidrug Therapy (MDT) or the Uniform 6 Doses MDT
Source: Front Immunol. 2018 May 14;9:915. doi: 10.3389/fimmu.2018.00915 (PMC5960669; doi:10.3389/fimmu.2018.00915)
Supplement: Supplementary file 1 [file table_1.DOCX]

**Supplementary Table 1. Comparisons of the positivity rates to anti-PGL-I, anti-LID-1 and anti-ND-O-LID serology among MB patients from R-MDT and U-MDT groups including and excluding BT and BB patients.**

| **R-MDT** | | | | **U-MDT** | | |
| --- | --- | --- | --- | --- | --- | --- |
| **BT, BB, BL, LL** | | **BL, LL** | ***p value*** | **BT, BB, BL, LL** | **BL, LL** | ***p value*** |
| **PGL-I** | | | | | | |
| **M0** | 70% (80/113) | 65% (74/113) | ns (0.73 ) | 74% (98/132) | 69% (92/132) | ns (0.68) |
| **M6** | 63% (70/111) | 57% (63/111) | ns (0.92) | 66% (80/121) | 56% (68/121) | ns (0.06) |
| **M12** | 46% (42/92) | 44% (41/92) | ns (1.76) | 51% (42/82) | 49% (40/82) | ns (0.38) |
| **1st year** | 40% (27/68) | 29% (20/68) | ns (0.07) | 55% (43/78) | 50% (39/78) | ns (0.41) |
| **2nd year** | 36% (15/42) | 36% (15/42) | ns | 39% (18/45) | 33% (15/45) | ns (0.43) |
| **LID-1** | | | | | | |
| **M0** | 88% (99/113) | 83% (94/113) | ns (0.41) | 88% (116/132) | 82%(109/132) | ns (0.11) |
| **M6** | 84% (93/111) | 74% (82/111) | p=0.03 | 86% (104/121) | 73% (89/121) | p=0.01 |
| **M12** | 79% (73/92) | 72% (66/92) | ns (0.75) | 80% (66/82) | 73% (60/82) | ns (0.86) |
| **1st year** | 62% (42/68) | 54% (37/68) | ns (0.89) | 78% (61/78) | 70% (55/78) | ns (0.84) |
| **2nd year** | 61% (26/42) | 55% (23/42) | ns (0.44) | 65% (30/45) | 56% (26/45) | ns (0.76) |
| **ND-O-LID** | | | | | | |
| **M0** | 73% (82/113) | 67% (76/113) | Ns (0.76) | 73% (97/132) | 70% (93/132) | ns (0.30) |
| **M6** | 63% (70/111) | 54% (60/111) | ns (0.08) | 62% (75/121) | 55% (66/121) | ns (0.12) |
| **M12** | 54% (50/92) | 47% (44/92) | ns (0.78) | 47% (39/82) | 43% (35/82) | ns (0.39) |
| **1st year** | 31% (21/68) | 28% (19/68) | ns (0.35) | 45% (35/78) | 42% (33/78) | ns (0.10) |
| **2nd year** | 17% (7/42) | 14% (6/42) | ns (0.38) | 30% (14/45) | 29% (13/45) | ns (0.41) |

R-MDT: regular MDT, U-MDT: uniform MDT; different time points of sera collection and testing: before MDT (M0/month zero), 6-12 months after the start of MDT (M6- M12) and at the first and second year after the conclusion of treatment (1^st^ year, 2^nd^ year). BT: borderline tuberculoid, BB: borderline-borderline, BL: borderline lepromatous, LL; lepromatous leprosy.

| **PGL-I** | | | | | | |
| --- | --- | --- | --- | --- | --- | --- |
| **R-MDT** | | | | **U-MDT** | | |
| **BT, BB, BL, LL** | | **BL, LL** | ***p value*** | **BT, BB, BL, LL** | **BL, LL** | ***p value*** |
| **M0** | (70%) 80/113 | (65%) 74/113 | *ns (0.7338 )* | (74%) 98/132 | (69%) 92/132 | *ns (0.676)* |
| **M6** | (63%) 70/111 | (57%) 63/111 | *ns (0.919)* | (66%) 80/121 | (56%) 68/121 | *ns (0.056)* |
| **M12** | (46%) 42/92 | (44%) 41/92 | *ns (1.761)* | (51%) 42/82 | (49%) 40/82 | *ns (0.377)* |
| **1st year** | (40%) 27/68 | (29%) 20/68 | *ns (0.068)* | (55%) 43/78 | (50%) 39/78 | *ns (0.411)* |
| **2nd year** | (36%) 15/42 | (36%) 15/42 | *ns* | (39%) 18/45 | (33%) 15/45 | *ns (0.431)* |
| **LID-1** | | | | | | |
| **R-MDT** | | | | **U-MDT** | | |
| **BT, BB, BL, LL** | | **BL, LL** | ***p value*** | **BT, BB, BL, LL** | **BL, LL** | ***p value*** |
| **M0** | (88%) 99/113 | (83%) 94/113 | *ns (0.411)* | (88%) 116/132 | (82%) 109/132 | *ns (0.113)* |
| **M6** | (84%) 93/111 | (74%) 82/111 | *p=0.03* | (86%) 104/121 | (73%) 89/121 | *p=0.01* |
| **M12** | (79%) 73/92 | (72%) 66/92 | *ns (0.755)* | (80%) 66/82 | (73%) 60/82 | *ns (0.856)* |
| **1st year** | (62%) 42/68 | (54%) 37/68 | *ns (0.887)* | (78%) 61/78 | (70%) 55/78 | *ns (0.840)* |
| **2nd year** | (61%) 26/42 | (55%) 23/42 | *ns (0.441)* | (65%) 30/45 | (56%) 26/45 | *ns (0.756)* |
| **ND-O-LID** | | | | | | |
| **R-MDT** | | | | **U-MDT** | | |
| **BT, BB, BL, LL** | | **BL, LL** | ***p value*** | **BT, BB, BL, LL** | **BL, LL** | ***p value*** |
| **M0** | (73%) 82/113 | (67%) 76/113 | *ns (0.757)* | (73%) 97/132 | (70%) 93/132 | *ns (0.301)* |
| **M6** | (63%) 70/111 | (54%) 60/111 | *ns (0.08)* | (62%) 75/121 | (55%) 66/121 | *ns (0.12)* |
| **M12** | (54%) 50/92 | (47%) 44/92 | *ns (0.783)* | (47%) 39/82 | (43%) 35/82 | *ns (0.394)* |
| **1st year** | (31%) 21/68 | (28%) 19/68 | *ns (0.353)* | (45%) 35/78 | (42%) 33/78 | *ns (0.104)* |
| **2nd year** | (17%) 7/42 | (14%) 6/42 | *ns (0.381)* | (30%)14/45 | (29%) 13/45 | *ns (0.409)* |
